# A high-throughput and sensitive method to measure Global DNA Methylation: Application in Lung Cancer

**DOI:** 10.1186/1471-2407-8-222

**Published:** 2008-08-03

**Authors:** Anthony Anisowicz, Hui Huang, Karen I Braunschweiger, Ziying Liu, Heidi Giese, Huajun Wang, Sergey Mamaev, Jerzy Olejnik, Pierre P Massion, Richard G Del Mastro

**Affiliations:** 1 Molecular Therapeutics Division, AmberGen Incorporated, Waltham, Massachusetts, USA; 2 Division of Allergy, Pulmonary and Critical Care Medicine, Vanderbilt Ingram Cancer Center, Nashville, Tennessee, USA

## Abstract

**Background:**

Genome-wide changes in DNA methylation are an epigenetic phenomenon that can lead to the development of disease. The study of global DNA methylation utilizes technology that requires both expensive equipment and highly specialized skill sets.

**Methods:**

We have designed and developed an assay, *CpG*lobal, which is easy-to-use, does not utilize PCR, radioactivity and expensive equipment. *CpG*lobal utilizes methyl-sensitive restriction enzymes, HRP Neutravidin to detect the biotinylated nucleotides incorporated in an end-fill reaction and a luminometer to measure the chemiluminescence. The assay shows high accuracy and reproducibility in measuring global DNA methylation. Furthermore, *CpG*lobal correlates significantly with High Performance Capillary Electrophoresis (HPCE), a gold standard technology. We have applied the technology to understand the role of global DNA methylation in the natural history of lung cancer. World-wide, it is the leading cause of death attributed to any cancer. The survival rate is 15% over 5 years due to the lack of any clinical symptoms until the disease has progressed to a stage where cure is limited.

**Results:**

Through the use of cell lines and paired normal/tumor samples from patients with non-small cell lung cancer (NSCLC) we show that global DNA hypomethylation is highly associated with the progression of the tumor. In addition, the results provide the first indication that the normal part of the lung from a cancer patient has already experienced a loss of methylation compared to a normal individual.

**Conclusion:**

By detecting these changes in global DNA methylation, *CpG*lobal may have a role as a barometer for the onset and development of lung cancer.

## Background

The functional role of DNA methylation includes maintaining the stability of chromosomes, silencing repetitive sequences, arresting the deleterious effects of integrated foreign DNA and controlling gene expression [1]. Genome-wide loss of the methyl group at 5-methyl cytosines (hypomethylation) leads to the destabilization of the DNA [2]. Global DNA hypomethylation has been observed to be one of the earliest molecular abnormalities described in human neoplasia [3,4]. This biological phenomenon could be exploited to gain an insight into the mechanism of action of DNA methylation to determine how it plays a role not only in disease but also in aging, diet and efficacy of drugs.

Several technologies have been developed to measure the methyl content of the genome. In general the techniques have been focused around the use of methodologies that can quantitate the 5-methyl cytosines using reversed-phase high performance liquid chromatography (RP-HPLC), two dimensional thin layer chromatography (2D-TLC), high performance liquid chromatography-mass spectrometry (HPLC-MS), high performance capillary electrophoresis (HPCE) and liquid chromatography-electrospray ionization-tandem mass spectrometry (LC-ESI-MS/MS) [5-10]. These approaches represent the gold standards of measuring global DNA methylation but lack the capacity for high-throughput sample handling, require expensive equipment to analyze the material and involve highly specialized skill sets. Other approaches that have been developed to quantitate the global 5-methyl cytosines include radio-labeling the CpG sites using M.SssI methyltransferase, methyl-C antibody, pyrosequencing and methyl sensitive restriction enzymes [11-17]. We have developed an approach that has been adapted from a method that uses methyl sensitive restriction enzymes. These enzymes have been used to define the methylation status of both the whole genome and specific regions using a host of technologies [14-17]. In this paper we present *CpG*lobal, a non-radioactive, non-PCR, high-throughput approach to measure global DNA methylation. It describes how *CpG*lobal is performed in a microtiter plate, which enables multiple samples to be analyzed simultaneously. Furthermore, this paper illustrates how the assay utilizes biotinylated nucleotides to provide a highly accurate and reproducible method that can measure global DNA methylation using only 100 ng of genomic DNA per reaction. In addition, we demonstrate that *CpG*lobal is an excellent alternative to HPCE, one of the gold standard technologies.

*CpG*lobal has been employed to study the role of global DNA methylation in lung cancer to understand further the biology of this disease. It is the world's most common fatal cancer with an overall survival rate of 15% over 5 years [18]. This dismal outcome can be attributed to the natural history of the disease where in its early stages it is asymptomatic and in the latter stages patients present with non-specific symptoms [19]. In order to gain further insight into the natural history of this disease we measured global DNA methylation in a set of lung cancer cell lines that represented all the stages of this disorder as well as in 20 paired normal/tumor from patients diagnosed with Non-small cell lung cancer (NSCLC). The resultant data showed that there was an increase in hypomethylation observed with tumor progression as well as in the normal part of the lung from cancer patients.

## Methods

### Measurement of Global DNA Methylation

To quantitate the amount of DNA methylation in any genome 100 ng of a sample was aliquoted nine times into a 96 well white Microfluor 2 plate (Thermo Electron, Waltham, MA). The genomic DNA in the first three wells was digested with 5 units of a methyl-sensitive restriction enzyme such as HpaII (New England BioLabs, Beverly, MA), the DNA in the second three wells was digested with 5 units of the methyl-insensitive restriction enzyme MspI (New England BioLabs, Beverly, MA) to normalize the data when calculating the Global DNA Methylation Index (GDMI), and the third set of triplicates was for buffer only (NEBuffer1 – New England BioLabs, Beverly, MA). All reactions were performed in a total volume of 30 μl. Prior to incubation the 96 well microtiter plate was sealed with Adhesive PCR Foil (ABgene Inc., Rochester, NY) using an ALPS 300™ (ABgene Inc., Rochester, NY), spun briefly and placed in an air incubator for 3 hours at 37°C. After incubation, the plate was spun briefly and the Adhesive PCR Foil removed. The digestion of DNA was followed by an end-fill reaction where 20 μl of biotinylation buffer containing Biotin-11-dCTP and Biotin-11-dGTP (Perkin Elmer, Boston, MA) and Sequenase (USB Corporation, Clevland, OH) in 40 mM Tris-HCl, pH 7.5, 20 mM Tris-HCl, 50 mM NaCl) was added. The final concentration of biotinylated dCTP and dGTP and Sequenase used per well was 0.1 μM and 0.1 units respectively. The plate was sealed, spun briefly and placed into an air incubator for 30 minutes at 37°C. After the incubation period the plate was spun briefly, the Adhesive PCR Foil removed and 100 μl of Reacti-Bind™ DNA Coating Solution (Pierce, Rockford, IL) was added to each well. After mixing, the plate was once again sealed and placed on an orbital platform shaker (Lab-Line Instruments Inc., Melrose Park, IL) and shaken at 150 RPM at room temperature overnight. The solution in the wells was removed and the wells were washed 4 times with TBS (10 mM Tris-HCL pH 8.0, 150 mM NaCl). The biotin was detected using HRP Neutravidin (Pierce, Rockford, IL) and the DNA Detector kit (KPL, Gaithersburg, MD). Briefly, 200 μl of the Detector Block Solution (KPL, Gaithersburg, MD) was added to each well and the plate was incubated for 30 minutes at room temperature. After removal of the Detector Block Solution, 150 μl of Detector Block Solution containing 0.5 μg/ml (1:2000 dilution) of HRP Neutravidin was added to each well and the plate was incubated at room temperature for 30 minutes. The Detector Block/HRP Neutravidin solution was removed and the wells washed 5 times with 1xBiotin wash solution (KPL, Gaithersburg, MD). After the final wash 150 μl of LumiGlo^® ^chemiluminescence substrate (KPL, Gaithersburg, MD) was added to each well. After 2 minutes the luminescence emitted from each well was quantitated by a Wallac Envision 2100 multilabel reader (Perkin Elmer, Boston, MA).

### Generation of fully methylated lambda DNA

To determine the analytical sensitivity of *CpG*lobal the entire genome of Lambda DNA was methylated using M.SssI (New England BioLabs, Beverly, MA), a bacterial CpG methylase, which methylated all the cytosine residues that resided within a CpG dyad. Fifty micrograms of lambda genomic DNA was methylated with M.SssI for 4 hours at 37°C. The salt and enzyme were removed from the methylated lambda DNA using QIAEX II (Qiagen, Valencia, CA). To determine whether the lambda DNA was fully methylated 1 μg of the product was aliquoted and digested with MspI (methyl insensitive restriction enzyme) and another microgram with HpaII (a methyl sensitive restriction enzyme). The digested products were analyzed by gel electrophoresis. No fragments were observed in the HpaII digested lane. The expected size bands were measured in the MspI digested lane.

### Linearity of CpGlobal using lambda DNA

To determine the efficiency of digestion and end-fill reactions for an amount of lambda genomic DNA, we used the methyl-insensitive restriction enzyme MspI (CCGG) for which there were 328 restriction sites in the genome. The genomic DNA was aliquoted six times into a 96 well microtiter Microfluor 2 White plate. One set of triplicates were digested with MspI (New England BioLabs, Beverely, MA), the second set was treated with buffer only (NEBuffer2 – New England Biolabs, Beverely, MA). A range of DNA concentrations (25 ng, 12.5 ng, 6.25 ng, 3.12 ng) were assayed to determine the linearity of *CpG*lobal. The assay and the quantitation of the chemiluminescence were performed as described above. The net luminescence per DNA concentration was plotted and 25 ng of lambda DNA was determined to provide the appropriate signal to noise level for the next set of experiments.

### Accuracy of CpGlobal using lambda DNA

To ascertain the accuracy of *CpG*lobal and determine the best methyl-sensitive restriction enzyme that could be applied to this assay, a series of mixtures with increasing ratios of unmethylated to methylated Lambda DNA was generated. The series of mixtures increased by 5% such that the range of methylated to unmethylated produced a broad spectrum of DNA methylation densities that varied from 100% to 0% methylated. Once the mixtures were generated, 25 ng of each DNA mix was aliquoted nine times into a 96 well microtiter Microfluor 2 White plate. To quantitate the amount of methylated Lambda DNA in each mixture the first three aliquots were digested with 5 units of a methyl-sensitive restriction enzyme (AciI, BstUI HpaII, HinP1I, HypCH4IV) (New England BioLabs, Beverely, MA), the second three aliquots were digested with 5 units of the methyl-insensitive restriction enzyme MspI (New England BioLabs, Beverely, MA) and the third three aliquots were mixed with buffer only. The following NEB buffers (New England Biolabs, Beverely, MA) were used for different enzymes: NEBuffer1 (HpaII, MspI, HpyCH4IV), NEBuffer2 (BstUI and HinP1I) and NEBuffer3 (AciI). The assay and the quantitation of the chemiluminescence were performed as described above except for BstUI. This enzyme is a blunt end cutter and instead of using Sequenase during the end-fill reaction 0.1 units Klenow (New England BioLabs, Beverely, MA) was used in its stead. Here, the 3'-5'exonuclease activity of the Klenow was utilized to chew back into the blunt end-fragment and then the 5'-3'exonuclease activity would incorporate the biotinylated nucleotides.

### Quantitative Range of CpGlobal using human genomic DNA

The linear range of the *CpG*lobal assay for human genomic DNA was determined by measuring global DNA methylation in human male genomic DNA isolated from whole blood (Novagen, San Diego, CA). A range of DNA concentrations (100 ng, 50 ng, 25 ng, 12.5 ng, 6.25 ng and 3.125 ng) were assayed to determine the linearity of *CpG*lobal. One set of triplicates were digested with HpaII (New England BioLabs, Beverely, MA), the second set with MspI (New England BioLabs, Beverely, MA) and the third set was treated with buffer only (NEBuffer1 – New England Biolabs, Beverely, MA). The assay and the quantitation of the chemiluminescence were performed as described above.

### Cell Culture and DNA Isolation

All cell lines were purchased from ATCC (Manassas, VA) and were grown according to the manufacturer's recommendations. The cell lines that were used to compare the *CpG*lobal with High Performance Capillary Electrophoresis (HPCE) were SW48 (CCL-231), LoVo (CCL-229), HT-29 (HTB-38) and NCI-H69 (HTB-119D). Assessment of the global DNA methylation was performed on Non-Small Cell Lung Cancer tumor cell lines from various stages of lung cancer. These included a normal lung NL20 (CRL-2503), Stage I NCI-H1703 (CRL-5889), Stage II NCI-H522 (CRL-5810), Stage IIIa NCI-H1993 (CRL-5909), Stage IIIb NCI-H1944 (CRL5907) and Stage IV metastatic liver NCI-H1755 (CRL-5892). All cells were grown in 75 cm^2 ^cell culture flasks (Corning Incorporated, Corning, NY) in 5% CO_2 _at 37°C. Cells were harvested when they had reached 90% confluency. DNA was isolated using the Blood and Cell Culture DNA Midi Kit (Qiagen, Valencia, CA). The purity and concentration of the DNA was measured using a DU650 spectrophotometer (Beckman Coulter, Fullerton, CA). The quality of the DNA was determined by loading 200 ng on a 1% agarose gel to inspect for any degradation.

### Lung Tissue samples

Twenty fresh frozen tissues samples, including lung tumors and areas of non involved lung, were obtained from a repository of biological specimens through the lung cancer Specialized Program of Research Excellence (SPORE) at the Vanderbilt Ingram Cancer Center. For each specimen, a quality control of the tissue specimen was obtained from hematoxylin and eosin stained tissue section on adjacent tissue and reviewed by a pathologist prior to analysis. De-identified clinical data elements were shared in compliance of the health insurance portability and accountability regulations. This study was approved by the local Institutional Review Board of both institutions.

### Normal lung DNA

Normal lung DNA from young male accident victims was purchased from BioChain Institute, Inc., Hayward, CA.

### DNA isolation

Approximately 0.1 gm lung tissue from biopsies of paired tumor and normal tissue from NSCLC patients was frozen in liquid nitrogen. The tissue was pulverized to a fine powder using a freezer mill 6750 (SPEX SamplePrep, Metuchen, NJ) according to the manufacturer's recommendations. DNA was prepared using the Blood and Cell Culture DNA midi kit (Qiagen, Chatsworth, CA). The DNA was checked for concentration and purity by reading the absorbance at 260 and 280 nM and was further tested for intactness by running 100 ng on a 1% agarose gel.

### Linear Regression Analysis

Linear regression analyss was performed using EXCEL Analysis ToolPak Regression Analysis. In each case presented single predictor variable was used. R square, Adjusted R square, intercept, coefficient, standard error and F significance were generated by standard Summary Report. Residual Plots and LineFit Plots were used to check for the linearity of the regression.

### Calculation of Global DNA Methylation Index (GDMI)

To determine the net luminescence, the average value for the no enzyme control was subtracted from the individual enzyme values. To calculate the global DNA methylation index (GDMI), the individual net luminescence values for the methylation sensitive enzyme were divided by the average Msp I net luminescence. All calculations were performed in Microsoft Excel.

### Statistical Analysis

Both means and medians were calculated when comparing group differences among Normal Non-Disease, Normal Disease and Tumor samples. Only medians were used when the distributions of continuous variables were significantly skewed by small sample sizes such as different histopathological groups. Due to the potential compromise of parametric assumption, non-parametric Wilcoxon/Kruskal-Wallis tests were applied to evaluate the statistical significances for all analysis. P-value <= 0.05 was considered as statistically significant and p-value > 0.05 and <= 0.10 was regarded as border-line significant. All analyses were conducted using JMP5.1 (SAS Institute, Inc., Cary, NC).

## Results

### Design of CpGlobal

**Figure 1:**
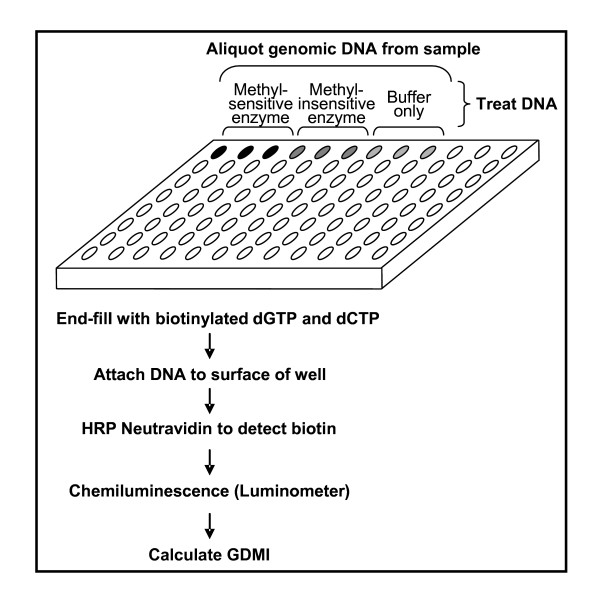
Schematic showing design of *CpG*lobal.

*CpG*lobal was designed to incorporate several key features: easy to use, did not require radioactivity or PCR, functioned in a 96-well microtiter plate and utilized equipment that most laboratories possessed. The design was adapted from a method that utilizes methyl-sensitive restriction enzymes [14], which when applied to genomic DNA would produce a set of digested fragments that could be quantitated and translated into an amount of DNA methylation present in the genome. We have modified the approach by performing all the steps in one 96 well microtiter plate. Therefore, once the genomic DNA from a sample was aliquoted into the well of the microtiter plate it remained in that location through the four stages of treatment: digestion, end-fill with biotinylated nucleotides, attachment to the surface of the well and chemiluminescence. The methyl-insensitive restriction enzyme MspI, the isoschizomer of HpaII, was used in the design to normalize the data collected from the methyl-sensitive restriction enzymes. This step aided in the removal of any intrinsic variations introduced through slight differences in DNA concentrations, and digestion and end-fill reactions. As such a global DNA methylation index (GDMI) was calculated. The arrangement of the DNA in the 96 well microtiter plate was designed so that up to 10 samples per plate could be analyzed (Figure 1). The final step in the assay was the measurement of luminescence through the use of a luminometer. The end-product was an assay designed for an operator to easily manage the measurement of global DNA methylation in multiple samples, without the need for radioactivity, PCR and expensive equipment.

### Accuracy of CpGlobal

Based on the design of the assay we began with the application of *CpG*lobal on Lambda DNA with the aim to determine which methyl-sensitive restriction enzyme was the best indicator in measuring global DNA methylation. Two species of lambda DNA were created: one that was unmethylated and the other that was *in vitro *methylated using M.SssI, a CpG methylase. Five 4 base pair methyl-sensitive restriction enzymes, AciI (CCGC), BstUI (CGCG), HpaII (CCGG), HinP1I (GCGC) and HypCH4IV (ACGT), were chosen based on their frequency within the Lambda genome and the core recognition sequence site. These enzymes were used to digest a series of Lambda DNA mixtures, which consisted of 21 points that extended from 100% to 0% methylated in 5% intervals. GDMI was generated for each 5% increment, which was compared against the theoretical methylation level. A linear regression analysis was performed for each methyl-sensitive restriction enzyme (Table 1). We observed a highly significant correlation of GDMI with the theoretical methylation levels using HinP1I, HpaII and HypCH4IV. Thus, under these conditions the use of any one of these three methyl-sensitive restriction enzymes would be an effective indicator for global DNA methylation.

**Table 1:** Linear regression analysis to determine which methyl-sensitive restriction enzymes were effective in *CpG*lobal.

**Enzyme**	**Theoretical Regression**	**Actual Regression**	**Adjusted R Square**	**Standard Error**	**F significance**
AciI	y = 1.57-1.57x	y = 0.45-0.46x	0.86	0.06	1.11E-09
BstUI	y = 0.48-0.48x	y = 0.09-0.11x	0.87	0.01	4.39E-10
HinP1I	y = 0.66-0.66x	y = 0.95-0.89x	0.97	0.05	1.24E-15
HpaII	y = 1.00-1.00x	y = 0.94-0.95x	0.96	0.06	4.46E-15
HpyCH4IV	y = 0.44-0.44x	y = 0.74-0.73x	0.97	0.04	7.67E-17

Theoretical regression formulas were calculated based on the number of recognition sites in lambda DNA for each of the methyl-sensitive restriction enzymes-AciI: 516, BstUI: 157, HinP1I: 215, HpaII and the isoschizomer MspI: 328, and HpyCH4IV: 143. The Actual regression formulae were calculated based on the regression analysis using the theoretical methylation level as a predictor for the results from *CpG*lobal.

### Application of CpGlobal in human genomic DNA

**Figure 2:**
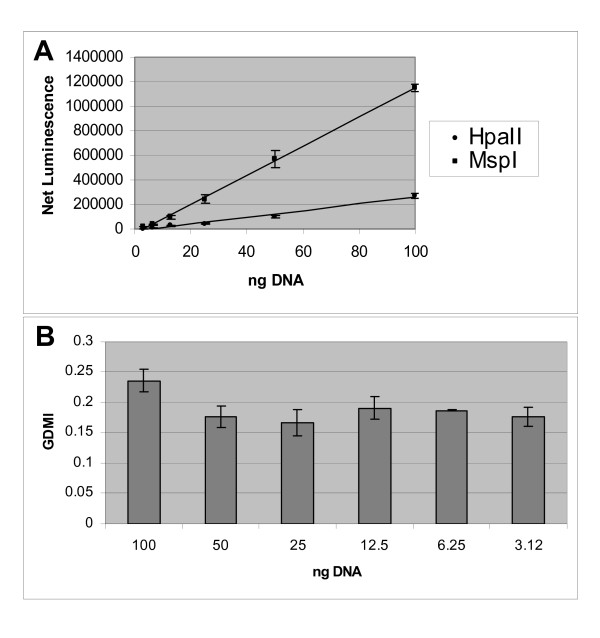
**Assessment of *CpG*lobal using human genomic DNA isolated from whole blood**. A) Linearity of CpGlobal for different amounts of lymphocyte DNA. Twofold serial dilutions of genomic DNA from 100 ng down to 3.12 ng digested with HpaII or MspI. Net Luminescence of the no enzyme control is subtracted from the net luminescence of HpaII and MspI and plotted versus concentration of DNA. The points fit with a linear regression (HpaII R^2 ^= 0.982 and Msp I R^2 ^= 0.999). B) Net HpaII luminescence was divided by net MspI luminescence to get global DNA methylation index (GDMI) and plotted versus DNA concentration.

In order to quantitate global DNA methylation in the human genome it was essential that the assay operates within the linear range. In particular, it was important to determine if the performance of a methyl-sensitive and the methyl-insensitive restriction enzyme was linear with DNA concentration as the net luminescence generated from the assay would be used to calculate the GDMI. Subsequently, *CpG*lobal was applied to human genomic DNA isolated from whole blood. Using a two-fold serial dilution that ranged from 100 ng to 3.125 ng, the DNA for each concentration was digested in triplicate with HpaII, MspI and treated with buffer only. The net luminescence from each experiment was plotted against DNA concentration (Figure 2a). The results demonstrated a linear regression that correlated highly with DNA concentration and net luminescence (R^2 ^HpaII = 0.982, R^2 ^MspI = 0.999). The GDMI was calculated for human whole blood DNA and was plotted against DNA concentration. The GDMI was moderately consistent with DNA concentration (Figure 2b). We chose to use 100 ng of human genomic DNA in all the following experiments because the signal to background ratio was at least > 10 fold.

### Comparison of CpGlobal with High-performance capillary elecrophoresis (HPCE)

HPCE is viewed as one of the gold standard technologies when measuring the global genomic content of 5-methylcytosine [8]. We applied *CpG*lobal to calculate the GDMI from a selection of cancer cell lines to determine whether a strong correlation existed between these two technologies. We measured the global DNA methylation in four cancer cell lines (SW48, LoVo, HT-29 and H69), which varied in methyl content from high to low as determined by HPCE. HpaII and MspI were utilized on 100 ng of cell line genomic DNA to calculate the GDMI. The HPCE results, extracted from the publication by Paz et al [20] were compared with the calculated GDMIs from the four cell lines. The *CpG*lobal results presented with a good linear fit when compared to the HPCE data. In addition, we observed that the number of unmethylated HpaII sites had an inverse linear relationship with the total number of methylated cytosines (data not shown).

The linear fit for this correlation was calculated:

HPCE = -6.2 × GDMI + 7.18

Adjusted R^2 ^= 0.77

Residual error = 0.36.

Considering that the HPCE data were taken from a publication to create the correlation, these results demonstrated that *CpG*lobal could be utilized as an alternative technique to one of the gold standard technologies.

### Application of CpGlobal to lung cancer lines

**Figure 3:**
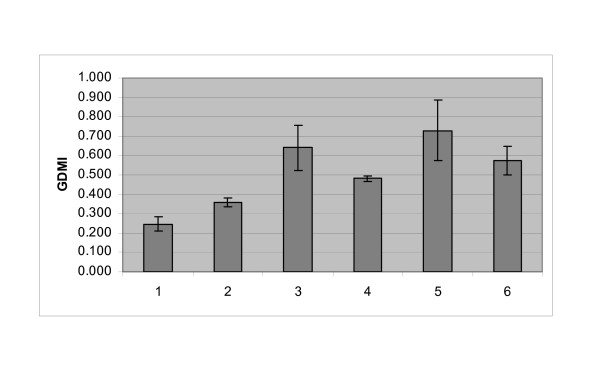
**Measurement of global DNA methylation in a set of lung cancer cell lines that represents the various stages of the disease**. 100 ng genomic DNA was digested with HpaII and MspI and the GDMI calculated: Lane 1, NL20 (normal lung); lane 2, NCI-H1703 (Stage 1 NSCLC); lane 3, NCI-H522 (Stage 2 NSCLC); lane 4, NCI-H1993 (stage 3A NSCLC); lane 5, NCI-H1944 (Stage 3B NSCLC); lane 6, NCI-H1755 (Stage 4 NSCLC).

We directed the assay toward measuring the association of global DNA methylation with the natural history of lung cancer. Lung cancer is measured in 5 stages, which range from 0 to IV [21]. We measured the GDMI, using the HpaII methyl-sensitive restriction enzyme, in epithelial cell lines that represented the full extent of the disease: Normal lung, Stage I, Stage II, Stage IIIa, Stage IIIb and Stage IV metastatic liver. The results indicated that there was an increase in global DNA hypomethylation that was associated with the progressive development of the tumor (Figure 3). The greatest loss of methylation was observed in the Stage IIIb cell line and the least was measured in the Stage I. Compared with the normal epithelial cell line, this represented a loss of 5-methylcytosines that ranged from 10%, in an early stage tumor, to 50% in a late stage cancer. These data demonstrated the quantitative capacity of *CpG*lobal to measure changes in global DNA methylation in a cancer as it progresses from early to late stage and metastasis.

### Global DNA methylation levels in normal tissue and lung cancer

We measured the GDMI using *CpG*lobal in lung DNA from 20 patients diagnosed with Non Small Cell Lung Cancer (NSCLC) (Table 2) and 12 normal individuals to ascertain whether global DNA hypomethylation is a prominent phenomenon in this disease. DNA from three types of lung tissues was analyzed: Normal Non-Disease from normal individuals, Normal Disease (pathological and histological defined normal) paired with Tumor from each cancer patient. The GDMI was measured in the lung DNAs using the methyl-sensitive restriction enzymes HinP1I, HpaII and HypCH4IV. A general trend was observed when the median GDMI was calculated for all three methyl-sensitive enzymes (Table 3a); an increase in the median GDMI was measured with lowest levels detected in the Normal Non-Diseased and the highest in the Tumors (median GDMI – Normal Non-Disease < Normal Disease < Tumor). Nonparametric Wilcoxon/Kruskal-Wallis test was performed on the median GDMI data to determine whether the observed trend was statistically significant (Table 3b). The greatest difference in GDMI was between the Normal Non-Disease and Tumor (p < 0.054–0.004), which was observed in all three methyl-sensitive enzymes. However, only HinP1I and HpaII were able to detect a GDMI difference between the paired Normal Disease and Tumor (p = 0.064 and p = 0.068 respectively). Out of the three methyl-sensitive enzymes only HpaII was observed to distinguish between the Normal Non-Disease and Normal Disease with statistical significance (p = 0.039). These results suggested that the histological and pathological defined normal part of the lung from a cancer patient had altered global DNA methylation levels.

**Table 2:** Patient information

				**Pathology**				
					
**Patient ID**	**Age**	**Race**	**Gender**	**Tumor**	**Lymph Node**	**Metastasis**	**Stage**	**Differentiation**
1	64	White	Male	T2	N0	Mx	Stage IB	Non-well Diff
2	59	White	Female	T1	N0	M0	Stage IA	Non-well Diff
3	85	White	Female	T1	N0	M0	Stage IA	Non-well Diff
4	62	White	Male	T1	N0	Mx	Stage IA	Well Diff
5	62	White	Male	T1	N1	Mx	Stage IIB	Non-well Diff
6	53	White	Male	T1	N0	M1	Stage IA	N/A
7	68	White	Female	T1	N0	Mx	Stage IA	Well Diff
8	60	Black	Female	T1	N0	M0	Stage IA	Non-well Diff
9	54	White	Male	T2	N0	Mx	Stage IB	Non-well Diff
10	77	White	Female	T2	N0	M0	Stage IA	Non-well Diff
11	71	White	Female	T1	N2	M1	Stage IIIA	Well Diff
12	47	Black	Female	T3	N/A	N/A	Stage IIIA	N/A
13	57	White	Female	T4	N0	M0	Stage IB	Non-well Diff
14	65	White	Female	T1	N0	M0	Stage IA	Non-well Diff
15	77	White	Male	T2	N0	M0	Stage IB	Non-well Diff
16	82	White	Male	T1	N0	Mx	Stage IA	Non-well Diff
17	85	White	Male	T2	N0	Mx	Stage IB	Non-well Diff
18	52	White	Male	T2	N0	M0	Stage IB	Non-well Diff
19	63	White	Male	T2	N0	M0	Stage IA	Non-well Diff
20	56	White	Male	T2	N0	M0	Stage IB	Non-well Diff

**Table 3:** Median GDMI of normal non-disease, normal disease and the paired tumors for different  methyl-sensitive restriction enzymes

**A**			
**Median GDMI of normal non-disease, normal disease and the paired tumors for different methyl-sensitive restriction enzymes.**			

**Sample Type**	**HinP1 I**	**HpyCH4 IV**	**Hpa II**

Normal Non-Disease	0.163	0.304	0.293
Normal Disease	0.197	0.368	0.321
Tumor	0.216	0.427	0.379

**B**			

**Non-parametric tests of normal disease versus paired tumor, normal non-disease versus normal disease, and normal non-disease versus tumor for each of the methyl-sensitive restriction enzymes.**			

**Comparisons**	**HinP1 I**	**HpyCH4 IV**	**Hpa II**

Normal Disease vs Tumor	0.068*	0.201	0.064*
Normal Non-Disease vs Normal Disease	0.483	0.129	0.039**
Normal Non-Disease vs Tumor	0.005**	0.054*	0.004**

Numbers in bold with asterisks indicate statistical significance of Wilcoxon/Kruskal-Wallis tests with threshold of p <= 0.05 (**) or p <= 0.10 (*).

### Distinguishing Properties of CpGlobal in Lung Cancer

To investigate further the changes in global DNA methylation in lung cancer we separated the major histopathologic characteristics assigned to the cancer patient samples into three groups (Stage – 1A and 1B, grade of differentiation – well and non-well and tumor size – T1 and T2) and searched for any association of the GDMI with these characteristics in the Normal Disease and Tumor samples. Nonparametric Wilcoxon/Kruskal-Wallis test was performed using the median GDMI data, which was measured in the Normal Disease and Tumor samples. No statistically significant changes were observed in global DNA methylation from the Normal Disease tissue samples for any methyl-sensitive restriction enzyme when compared against the three groups. However, the median GDMI from the Tumor tissue samples was significantly associated with all three histopathologic characteristics. In particular this was observed for HinP1I (Stage 1A versus 1B), HypCH4IV and HpaII (well versus non-well differentiated) and HinP1I and HpaII (T1 versus T2) (Table 4). When the analysis was performed using the ratio of median GDMI Tumor/median GDMI Normal Disease the statistical significance was improved for some methyl-sensitive restriction enzymes and made worse for others when compared against the three histopathologic characteristics (Table 4). These analyses indicated that global DNA hypomethylation remained a persistent observation in lung tissue from patients diagnosed with NSCLC. Furthermore, *CpG*lobal could clearly distinguish a difference in the Tumor samples between Stage 1A and 1B, tumor size T1 and T2, and well differentiated and non-well differentiated tissue.

**Table 4:** Median GDMI stratified by different histopathologic characteristics for normal disease, tumor, and their ratios.

**Characteristics**	**Enzymes**	**Normal Disease**		**p-value**	**Tumor**		**p-value**	**Tumor/Normal Disease**		**p-value**
						
		**(Median)**			**(Median)**			**(Median)**		
**Stage**		Stage IA (n = 10)	Stage IB (n = 7)		Stage IA (n = 10)	Stage IB (n = 7)		Stage IA (n = 10)	Stage IB (n = 7)	
	HinP1I	0.20	0.15	0.22	0.18	0.26	0.005**	0.92	1.74	0.006**
	HypCH4IV	0.37	0.37	0.92	0.39	0.43	0.59	1.05	1.33	0.08*
	HpaII	0.31	0.32	0.49	0.35	0.44	0.13	1.20	1.32	0.19

**Grade of Differentiation**		Well Diff (n = 3)	Nonwell Diff (n = 15)		Well Diff (n = 3)	Nonwell Diff (n = 15)		Well Diff (n = 3)	Nonwell Diff (n = 15)	
	HinP1I	0.20	0.20	0.31	0.19	0.25	0.17	1.11	1.21	0.86
	HypCH4IV	0.37	0.37	0.77	0.29	0.44	0.10*	0.79	1.14	0.04**
	HpaII	0.31	0.32	0.68	0.28	0.41	0.01**	0.82	1.33	0.01**

**Tumor Size**		T1 (n = 10)	T2 (n = 8)		T1 (n = 10)	T2 (n = 8)		T1 (n = 10)	T2 (n = 8)	
	HinP1I	0.20	0.16	0.66	0.18	0.25	0.04**	0.99	1.47	0.04**
	HypCH4IV	0.37	0.35	0.59	0.39	0.42	0.70	1.01	1.15	0.12
	HpaII	0.32	0.32	0.62	0.32	0.43	0.08*	1.14	1.31	0.17

Numbers in bold with asterisks indicate statistical significance of non-parametric Wilcoxon/Kruskal-Wallis tests with a threshold of p <= 0.05 (**) or p <= 0.10 (*).

## Discussion

To actively study the role of global DNA methylation in the context of disease requires complex and expensive equipment and skill sets that compliment the technology. We have designed and developed a technique, *CpG*lobal, which is easy-to-use, cost-effective and used skill sets and equipment that were present in most molecular biology laboratories. The assay did not require radioactivity or involve PCR. We found that *CpG*lobal correlated significantly with HPCE, which was one of the gold standard technologies used to measure global DNA methylation. Considering that the HPCE global DNA methylation data was retrieved from a publication [20], where the cell culture conditions may not have been similar to our standards, these results exemplified the quality of *CpG*lobal to measure the methyl content of a genome.

The use of certain methyl-sensitive restriction enzymes in this assay provided *CpG*lobal with a level of accuracy and reproducibility in the measurement of global DNA methylation. The methyl-sensitive enzymes that worked consistently were HpaII and HinP1I. While the performance of HypCH4IV was not as effective in our assay as the other two methyl-sensitive restriction enzymes, the recognition sequence ACGT made it unique compared to HpaII and HinP1I, which were CpG rich sites. Therefore, such a mixture of methyl sensitive restriction sites allowed a greater assessment of the genome's global methyl content.

The application of *CpG*lobal in a 96 well microtiter plate enabled multiple samples to be analyzed at once. We routinely assayed each sample in triplicate. As such 10 samples were measured at a time. Yet, by performing the assay in duplicate up to 16 samples could be incorporated into the 96 well microtiter format. In addition, we have extended the use of the assay to function in a 384 well microtiter plate, which shared the same characteristics as the 96 well format (data not shown). Here, up to 64 samples could be analyzed at once. However, the application of robotics to dispense the liquids would be more practical in this format. While the data presented in this paper were obtained from the assay where 100 ng of genomic DNA per well was used, the results showed that using as little as 3 ng could be utilized. However, we have observed that the signal to background ratio diminishes as less DNA is used (data not shown).

The ability to access high quality tissue to perform these experiments was one of the limiting factors. While the tissue that was obtained from patients with lung cancer was highly defined and scored by experts that are knowledgeable in the histology and pathology of lung cancer, the normal tissue was obtained from car crash victims. To acquire detailed information regarding normal tissue is often difficult and limited. The variability in the tissue from accident victims can cause irreproducibility in global DNA methylation data. This may be reflected in the time and cause of death. In addition, it may explain why the median GDMI data from the Normal Non-Disease tissue were slightly higher than expected. The importance of collecting high quality tissue from normal individuals outside of blood is an on-going issue. A well-defined normal tissue bank, where sex and age matched samples could be obtained, which could go hand-in-hand with patient material, would be invaluable for diagnostic studies that seek the ability to use tissue beyond blood. An additional factor, which may have influenced the data, could be the purity of the tumor from the matched patient material. While the tumor was matched with normal it was not micro-dissected and so heterogeneity could have been an issue. Nevertheless, while the median GDMI for the tumor tissue was greater that the matched Normal Disease it was not as high as expected and this may have been attributed to heterogeneity. Given the difficulties faced in this study in acquiring truly well defined tissue, the data still remained convincing and strongly suggested *CpG*lobal was able to clearly discern differences in global DNA methylation from Normal Non-Disease and Normal Disease clinical samples.

The application of *CpG*lobal in a disease environment provided an opportunity to ascertain the value of the technology as a research tool. In particular, how global DNA hypomethylation contributed to the instability of the genome and in the transformation of a cell from normal to disease. Genome-wide loss in methylation has been observed as one of the earliest known molecular abnormalities in human neoplasias [3]. This would suggest that a better understanding of this biological phenomenon may provide an insight into the evolution of cancer [4]. In that regard, we have applied *CpG*lobal to lung cancer to delve deeper into understanding the natural history of this disease. Through the use of samples from patients diagnosed with NSCLC, two major observations from these studies were construed. The first, demonstrated that there was a statistically significant loss of global DNA methylation as the tumor progressed from Stage 1A to 1B (p = 0.08–0.006). At this early stage in the disease the only other characteristics for a pathologist to stage the lung cancer is size and grade of differentiation of the tumor. Both of these were associated with global DNA hypomethylation (p = 0.04 and p = 0.04 – 0.01 respectively). The second suggested that the histological and pathological defined normal part of the lung had incurred a loss in global DNA methylation that was statistically significant when compared with normal individuals (p = 0.039). Considering the small sample size in this study, and that by histopathologic standards the non tumor lung tissue from the cancer patients were graded as normal, global DNA hypomethylation appears to be a measurable change. Also, several studies have observed global DNA hypomethylation in other normal disease tissues such as colon, breast and ovary [22-24]. In addition, this phenomenon was determined to be a whole tissue circumstance rather than a cell-by-cell event [24-26]. It is plausible to speculate that a tissue-wide loss in global DNA methylation suggests that the entire lung is in a pre-neoplasia state. Continued global DNA hypomethylation could be one of the key factors in driving the cells to become malignant. However, how much global DNA hypomethylation is required before malignant transformation occurs would require a longitudinal prospective study to be performed.

Exploiting these global DNA methylation changes may be of value in screening asymptomatic individuals who are at risk in developing lung cancer. An initial practical application would be to measure the global DNA methylation levels of a large sample set that contained prospective patient material. One type of sample set that could be used is from a prospective nested case control study such as the one that was utilized to measure the methylation status of the promoters from 14 genes in proximal sputum samples. Here, methyl sensitive PCR (MSP) was the technique applied to these samples. The results showed that this technique could detect changes 18 months prior to diagnosis with a 64% sensitivity and specificity [27]. Application of *CpG*lobal to this type of material may improve the result through combining the results of both global DNA methylation and region specific analysis using MSP. Furthermore, it may provide information as to the association of global DNA methylation and the natural history of the disease before the presence of any clinical symptoms.

## Conclusion

*CpG*lobal is an easy-to-use technique to study the biological role of global DNA methylation in the cell. The technique can be used in almost any practical laboratory without the need for expensive equipment or highly specialized skill sets. In addition, *CpG*lobal can be utilized as a research tool to investigate the epigenetic phenomenon in disease, aging, diet, efficacy of drugs and in any other vertebrate or invertebrate genome where methylation is employed. The application of *CpG*lobal to measure the changes in global DNA methylation in lung cancer has demonstrated that there exists a distinguishing difference between Normal Non-Disease and Normal Disease tissue. Such a change should be examined further and exploited to understand the functional role of this epigenetic phenomenon with the potential aim of providing a diagnostic for asymptomatic individuals who are at risk of developing lung cancer.

## Abbreviations

GDMI: global DNA methylation index; HPCE: high performance capillary electrophoresis; NSCLC: Non small cell lung cancer.

## Competing interests

The authors AA, HH, HW, SM and RGDM are co-inventors on a patent entitled "Diagnosing Diseases by Detecting DNA Methylation Changes" (United States Patent Application 20070292866). RGDM was a full time employee of AmberGen from 2005 to 2007 and is now a full time employee of Invitrogen Corporation. RGDM holds no stock or shares in AmberGen Incorporated but holds shares in Invitrogen Corporation. The article-processing charge was paid for by AmberGen Incorporated. There are no other financial or non-financial competing interests to declare that pertain to this manuscript.

## Authors' contributions

AA, KIB and HG designed and performed the initial experiments with *CpG*lobal. ZL and SM isolated the DNA from the lung cancer cell lines and from the normal and tumor patient lung samples. AA performed all the experiments using *CpG*lobal to measure the global DNA methylation in the cell lines and patient lung samples. HH and HW performed the statistical analysis. JO was responsible for providing interpretation of the chemistry behind *CpG*lobal. HH and RGDM were responsible for the overall conception of *CpG*lobal. HH, PPM and RGDM were responsible the conception of the study and contributed to the preparation of this manuscript.

## Pre-publication history

The pre-publication history for this paper can be accessed here:


